# Characterization of Esocid Herpesvirus 1 (EsHV1) from Europe

**DOI:** 10.3390/pathogens14010045

**Published:** 2025-01-08

**Authors:** Mikael Leijon, Charlotte Axén, Fereshteh Banihashem, Tobias Lilja, Petter Tibblin, Björn David Persson

**Affiliations:** 1 Swedish Veterinary Agency, 751 89 Uppsala, Swedencharlotte.axen@sva.se (C.A.); fereshteh.banihashem@sva.se (F.B.); tobias.lilja@sva.se (T.L.); 2 Ecology and Evolution in Microbial Model Systems, EEMiS, Department of Biology and Environmental Science, Linnaeus University, 391 82 Kalmar, Sweden; petter.tibblin@lnu.se

**Keywords:** EsHV1, pike, *Esox lucius*, northern pike, blue spot disease, Alloherpesviridae, genomic characterization

## Abstract

During routine sampling of northern pike, a male *Esox lucius* with circular blue-metallic granular spots mainly located on the head and back was identified. Histological investigations presented multifocally thickened epidermis rich in basophilic large structures with a granulated rim and a dense, non-granulated center. Other organs showed no signs of infection. Ultrastructural analysis of the skin revealed three different types of herpes-like structures predominantly located within enlarged vacuoles. PCR analysis and NGS of dissected skin tissue verified the presence of EsHV1 DNA. In this study, we describe the first identification of EsHV1 in mainland Europe. In addition, for the first time, full sequences of both the DNA polymerase and terminase of the virus is available, thus allowing for an improved phylogenetic placement of EsHV1 within the *Alloherpesviridae* family. In addition to the EsHV1 infected pike, we also observed that 11.1% of the pike were affected by lymphosarcoma, a hyperplasia-disease caused by retroviruses. In conclusion, viral infections in pike are relatively common and likely have consequences for the local population dynamics.

## 1. Introduction

The northern pike, *Esox Lucius*, is a common predator in fresh and brackish waters located in northern Europe, Russia, Canada, and the northern USA. Within the river and lake ecosystems, this apex predator plays a critical role in regulating the populations of its prey [1]. In the Baltic Sea, a large brackish water system in northern Europe, the number of pike is declining rapidly, which is believed to be caused by habitat degradation and altered species interactions [2,3,4]. Pike grow to large sizes if allowed to—the largest pike ever caught was 150 cm long and weighed in at 28.4 kg [5]—with the females becoming significantly larger than the males. Spawning occurs during spring in shallow, usually flooded river areas, where a large female can release more than 300,000 eggs over the course of several hours [6]. Spawning may be very violent and provides an excellent opportunity for various pathogens to spread between individuals. Few of the offspring survive to adult age, as the fry is frequently preyed upon by other fish, insects, and birds [5].

Viral disease occurs in the pike in the wild, but to what extent it is difficult to estimate. The first virus described in pike was the pike fry rhabdovirus (PFRV), identified in 1959. The virus caused two distinctly different diseases: “head disease”, with hydrocephalus causing swelling of the head and neurological symptoms, and “red disease”, characterized by muscle and connective tissue hemorrhage, causing swelling and reddish areas on the body [7]. Both diseases are limited to fry or young fish and cause high mortality. In fact, pike is also highly susceptible to other rhabdoviruses such as viral hemorrhagic septicemia virus (VSHV) and infectious hematopoietic necrosis virus (IHNV), both present in Europe and a great threat to the salmonid fish farming industry [8]. In addition to rhabdoviruses, the pike is affected by lymphosarcoma, especially in early spring during spawning. During the 1960s, the source of the sarcomas was linked to a type C retrovirus [9,10,11,12]. Unfortunately, very little information is available, and all trials to isolate or grow the virus have failed, even though successful transmission studies have been published [13]. In addition to RNA viruses, DNA viruses have also been identified in pike, such as the iridovirus lymphocystis disease virus (LCDV) (family *Iridoviridae*) [14] and the alloherpesvirus, esocid herpesvirus 1 (EsHV1) (family *Alloherpesviridae*) [15,16]. Whereas the LCDV causes proliferative lesions of the skin and fins, EsHV1 causes blue–white metallic granular patches of up to 5 mm. Transmission electron microscopy shows characteristic naked herpesvirus particles with an icosahedral nucleocapsid [15,16]. Partial sequencing of the EsHV1 polymerase and terminase places the virus closest to the salmonid herpesviruses (SalHV) and acipenserid herpesvirus 2 (AciHV2) [15], with the highest amino acid identity for the DNA polymerase and terminase to SalHV1 (45.2% identical) and AciHV2 (52.2% identical).

In this study, we identified and characterized an EsHV1 infection of a male pike caught in Lervik, 45 km short north of Kalmar, Sweden. For EsHV1, characteristic spots were sampled and DNA was extracted for PCR analysis and next-generation sequencing (NGS). In addition, spotted skin and internal organs were investigated by histology. Through a combination of Illumina short-read sequencing and minion long-read sequencing directly from infected tissue, we managed to assemble approximately 27,000 nucleotides of the genome, covering the full sequence of the polymerase and terminase. In addition to EsHV1-infected pike, numerous individuals were identified with various degrees of lymphosarcoma. Thus, pike is seemingly often affected by viral infections likely because of its role at the top of the food chain. The implications of these infections for the local pike population should be further studied.

## 2. Materials and Methods

### 2.1. Sample Collection

The infected northern pike was identified during routine surveillance of the pike spawning migration in Lerviksbäcken (57°04′23.1″ N 16°31′18.3″ E), where anadromus pike migrate from the Baltic Sea in spring for spawning. This spawning location harbors one of the largest populations (500–3000 spawning adults annually) of anadromus pike in Kalmarsund and is extensively studied [2,17,18,19]. During three days in April of 2022, the pikes migrating for spawning were caught and investigated for signs of viral infections. The suspected EsHV1-infected pike was identified by the high number of blue-metallic spots. The pike was euthanized, and skin samples including the spots were secured in both 95% ethanol and RNA-later (Qiagen, Hilden, Germany) for extraction of nucleic acids. Additional tissue was fixed in 2.5% phosphate-buffered glutaraldehyde (Sigma-Aldrich, St Louis, MO, USA) for transmission electron microscopy (TEM). Finally, internal organs were fixed in 4% phosphate-buffered formalin (Sigma-Aldrich, St Louis, MO, USA) for histological investigations. No further samples were taken for fungal or bacterial analysis.

### 2.2. Extraction of Nucleic Acids and PCR Analysis

For both DNA and RNA extraction, the tissue was first homogenized using bead beating with 5 mm steel balls for 3 × 20 s using an MP FastPrep-24 (MP Biomedicals, Irvine, CA, USA). The DNA was subsequently isolated using The Gene Jet genomic DNA purification kit (Thermo Scientific, Waltham, MA, USA) including RNAse treatment. Even though the suspected virus was a herpesvirus, total RNA was also isolated using the TRIzol (Invitrogen, Waltham, MA, USA) method following the manufacturer’s instructions. During RNA extraction, the potentially contaminating DNA was digested using the TURBO DNA-free^TM^ kit (Invitrogen, Waltham, MA, USA).

The presence of EsHV1 was verified by PCR amplification using the PCR assays described by Freitas et al., 2016 [15]. For each assay, increasing annealing temperatures were used covering a range of 10 °C in 6 steps. Amplification of DNA fragments were analyzed by agarose gel electrophoresis using a 100-base pair (bp) (Thermo Fisher Scientific) or 1 kilo base pair (kbp) (Thermo Fisher Scientific) as a reference for size.

### 2.3. Histology

Fixed tissue pieces were embedded in paraffin, cut, and placed onto microscope slides. Sections were stained with hematoxylin–eosin (HE) before microscopic evaluation at 40–1000× magnification. The skin, kidney, spleen, liver, and heart were evaluated for signs of alteration in structural integrity and function. This included, for instance, vacuolation (nutritional status, liver), inflammation, infection, hemorrhage, degeneration/necrosis, regeneration, hypertrophy, hyperplasia, and presence of malignities. Regarding kidneys, the sample was obtained from the distal part of the organ to enable evaluation of both hematopoietic tissue (interstitium) and osmoregulatory structures (nephrons). The heart was sectioned longitudinally to allow for evaluation of the atrium, bulbus, valves, and ventricle.

### 2.4. Transmission Electron Microscopy

Samples were rinsed with 0.1 M PB for 10 min prior to 1 h incubation in 1% osmium tetroxide (TAAB Laboratories Equipment Ltd., Aldermaston, UK) in 0.1 M PB. After rinsing in 0.1 M PB, samples were dehydrated using increasing concentrations of ethanol (50%, 70%, 95% and 99.9%) for 10 min each step, followed by 5 min of incubation in propylene oxide (TAAB Laboratories Equipment Ltd.). The tissue samples were then placed in a mixture of Epon Resin (Ted Pella, Redding, CA, USA) and propylene oxide (1:1) for 1 h, followed by 100% resin and left o/n. Subsequently, samples were embedded in capsules in newly prepared Epon resin and left for 1–2 h, then polymerized at 60 °C for 48 h. The specimens were cut into semi-thin sections (1–2 microns), stained in Toluidine Blue, and examined in a LM. The block was trimmed, and ultrathin sections (60–70 nm) were cut in an EM UC7 Ultramicrotome (Leica, Wetzlar, Germany) and placed onto a grid. The sections were subsequently contrasted with 5% uranyl acetate and Reynold’s lead citrate and visualized with Tecnai™ G2 Spirit BioTwin transmission electron microscope (Thermo Fisher/FEI) at 80 kV with an ORIUS SC200 CCD camera (version 2.11.1404.0) and Gatan Digital Micrograph software version 2008 (both from Gatan Inc. Pleasanton, CA, USA).

### 2.5. DNA Sequencing

For Illumina short-read sequencing, the DNA was prepared for sequencing using a NEXTERA-XT kit (Illumina Inc. San Diego, CA, USA) according to the manufacturer’s instructions. The quality of the libraries obtained was assessed by the Agilent 2100 Bioanalyzer (Agilent Technologies. Santa Clara, CA, USA). Libraries were sequenced on a MiSeq Instrument (Illumina Inc. San Diego, CA, USA) using a Miseq Reagent Kit v3 in a 600-cycle paired-end run. For MinION long read sequencing, the concentration of the prepared DNA sample was measured with a Qubit (Invitrogen, Waltham, MA, USA) broad-range dsDNA assay, and 1 µg was used for sequence library preparation using the Ligation Sequencing Kit SQK-LSK110 (Oxford Nanopore, Oxford, UK) following the standard protocol. The sample was loaded on a MinION flowcell 9.4 and sequenced for 72 h using super-accurate base calling in Minknow (Oxford Nanopore, Oxford, UK). Approximately 336 k reads were produced with 265 megabases and a pass quality filter (Q10). All the Sanger sequencing was performed by Macrogen (Seoul, Republic of Korea).

### 2.6. Genome Assembly

The paired-end reads were quality trimmed using a Trimmomatic v 0.39 [20] with a sliding window of four nucleotides, and an average quality score of 15 was required. The trimmed reads together with the MinION long reads were subjected to hybrid assembly using SPAdes v 3.15.4 in standard mode [21]. The assembled contigs were then classified using DIAMOND v. 2.0.9 [22] with a database for classification, which was created using the NCBI nr database of GenBank release 248 and the corresponding NCBI taxonomy databases.

### 2.7. Genomic Annotation and Analysis

The assembled genome was annotated using PROKKA v.1.14.5 [23], and further annotations, identifying potential introns, were carried out using HMMGene 1.1 [24], except for the terminase, which was performed manually in accordance with published results (e.g., WWU01670). Tentative protein assignments of the CDs were carried out using the online BLASTp service of NCBI (https://blast.ncbi.nlm.nih.gov/Blast.cgi, accessed on 1 November 2024).

The CLC genomics workbench version 24.0.1 (Qiagen) was used to carry out maximum likelihood phylogeny. The maximum likelihood calculation used the neighbor-joining construction model with the Jukes–Cantor and WAG nucleotide and protein substitution models, respectively. The transition/transversion ration was set to 2.0, and 4 substitution rate categories were used. The gamma distribution parameter was set to 1. The bootstrap analysis utilized 100 replicates. The trees were rooted using human herpesvirus 3 (accession NP_040151 for the polymerase and NP_040153 for the terminase) as the outgroup.

## 3. Results

Macro-, micro-, and ultra-structure description of ESHV1 pathology.

**Figure 1:**
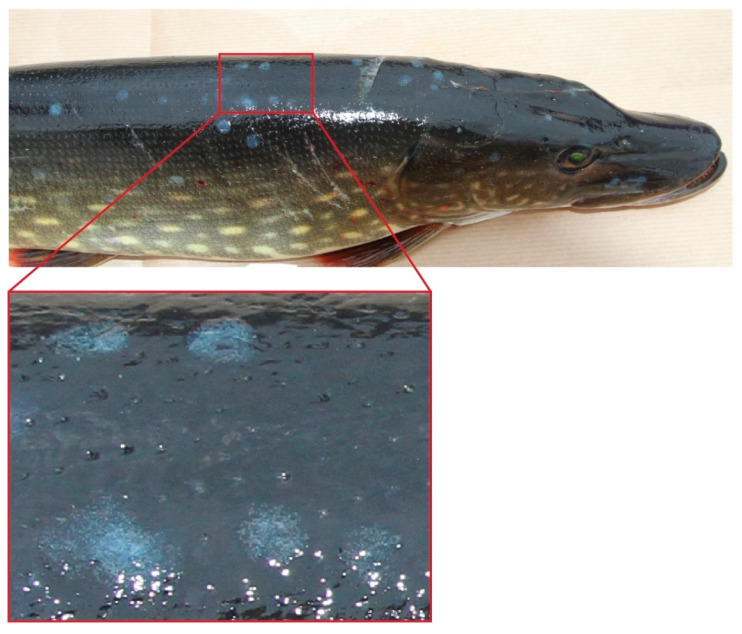
EsHV1-infected pike with the characteristic blue spots. Zoomed-in view of the spots highlights the punctuated blue-metallic pattern.

In total, 81 pikes were caught during the three days of sampling. Out of these 81 pikes, 10 had visible viral infections, 9 in the form of lymphosarcoma and 1 with EsHV1 that is described in this study. The male pike infected by EsHV1 was 53 cm long and of average fitness for the time of the year. Except for the presence of multiple blue metallic spots of about 5–8 mm in diameter with a punctuated pattern, no other external pathological observations were made (Figure 1). Most of the spots were present on the cranial third of the body. No spots were present on the fins. No macroscopic aberrancies were noted on internal organs, and the pike had been feeding recently, as undigested/partly digested prey was present in the stomach/intestine.

**Figure 2:**
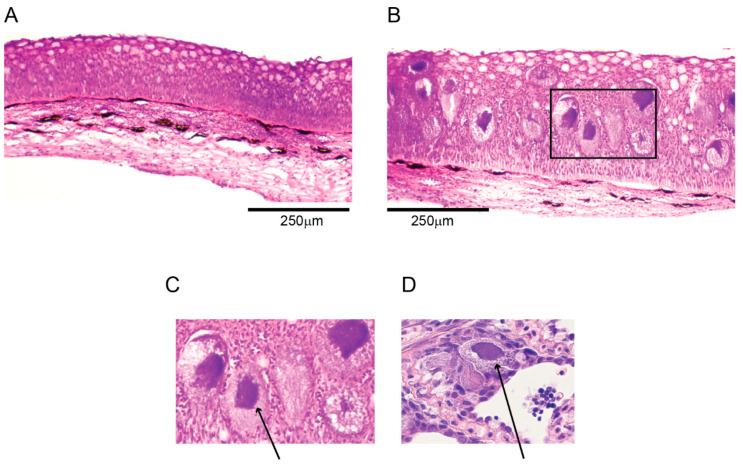
Histological investigation of pike skin with epidermis and superficial layer of spongious dermis. (**A**) Epidermis of normal thickness, rich in mucous cells. (**B**) EsHV1-infected skin with thickened epidermis. The black square indicates the position of panel (**C**). (**C**) Dense with hypertrophied, herpesvirus-infected epidermal cells (arrow). (**D**) GaHV-1-infected epithelial cell (arrow) in cod gill for comparison.

As the skin had been fixed in ethanol, the cell morphology and staining intensity were altered compared to what can be seen in histological sections of formalin-fixed skin, and thus, the skin sections could not be completely evaluated. The epidermis had a partially normal structure and was rich in mucus cells (Figure 2A), and partially consisting of multifocal areas of thickening. In the thickened areas, multiple large basophilic structures with granulated rims and dense, non-granulated centers could be seen, situated above the basal cell layer and beneath the superficial 3–4 cell layers, which were approximately 3–6 times the size of the superficial cells (Figure 2B). These structures were similar to previous descriptions of EsHV1 skin histopathology [15], as well as what we have observed in our laboratory for other herpesvirus infections like cod herpesvirus (GaHV-1, Figure 2D) [25], and were thus deemed as EsHV1-infected, hypertrophic cells with karyomegaly. Some of these hypertrophied cells contained a few condensed basophilic structures, potentially lymphocytes, at the rim (Figure 2A,B).

**Figure 3:**
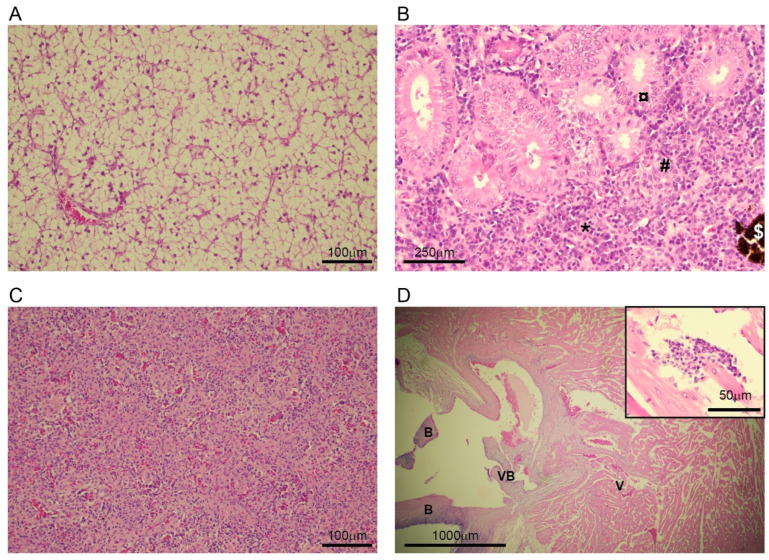
Histological investigation of internal organs. (**A**) Liver with fully vacuolated cells with mild nuclear condensation. (**B**) Kidney, normal nephron including glomerulus (#), tubuli (¤), and the interstitium (*). A melanomachrophage ($) center can be seen. (**C**) Spleen, normal appearance with low immune activity. (**D**) Heart at the base/ventriculobulbar junction. V, ventricle; B, bulbus; VB, ventriculo-bulbar valves. Overlapping image: small focus of myocarditis in the spongiosum.

None of the internal organs had alterations indicating a multisystemic viral infection. The liver showed extreme vacuolization. At low magnification, marginalized nuclei appeared to be pycnotic, but at higher magnification (≥20×), most nuclei had a normal “fried egg” appearance with a distinct nucleolus, and only some were pycnotic, indicating that fatty degeneration was not fully developed (Figure 3A). The distal kidney was rich in nephrons and the interstitium was dense, with various developmental stages of blood cells and melanomacrophage centers. A few tubuli had vacuolized epitheliums and mild neutrophil infiltration. In one tubulus, an ameboid-like cell was identified within the epithelial layer, and in one tubular lumen, a large multi-nucleate cell was present (Figure 3B). The spleen showed no signs of acute antigen reactivity, as the white pulp was normal with a low amount of white blood cells. There was a moderate amount of melanomacrophage centers (Figure 3C). In the heart, a small focus of myocardial inflammation was detected in the central spongiosum (Figure 3D).

**Figure 4:**
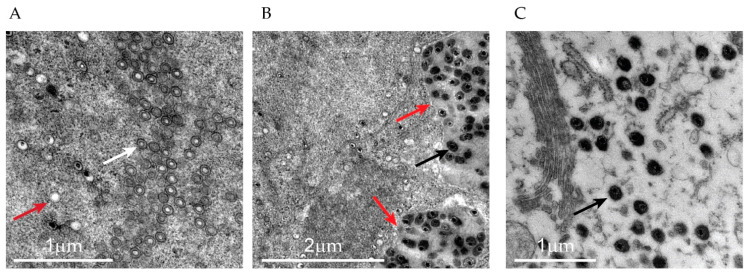
TEM analysis of infected skin. (**A**) Empty shells (A-capsids, red arrow) and mature capsids with clear centers (B-capsids, white arrow). (**B**) Mature C-capsids in large (black arrow), merged vacuoles (red arrow). (**C**) Higher-magnification image of mature C-capsids in close proximity to the Golgi apparatus and rough ER (black arrow).

TEM analysis of thin sections revealed areas of abundant virus production. Viral particles in various developmental stages were clearly observed, including empty shells (A-capsids, Figure 4A), mature capsids with a clear center (B-capsids, Figure 4A), and fully mature particles with electron-dense cores (C-capsids, Figure 4B,C). The mature capsids were largely located in large networks of cytoplasmic vacuoles.

### 3.1. PCR Verification of EsHV1

**Figure 5:**
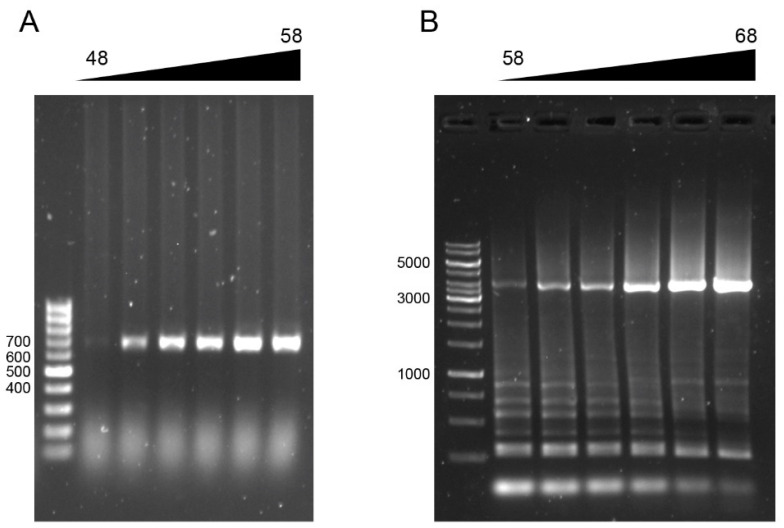
Verification of EsHV1 using PCR. (**A**) Amplification of the terminase of exon 2 (TERM). (**B**) Amplification of the region of the EsHV1 polymerase on terminase exon 1 (POLIN-TERM). In (**A**), a 100-base-pair marker was used for reference, and in (**B**), a 1-kilo base pair marker. Triangular shapes and numbers above the gel images indicate annealing temperatures increasing in 2 °C increments.

To verify the presence of EsHV1, we used the primers published by Freitas et al. in 2016 [15]. They designed a PCR assay consisting of four primer pairs binding into the polymerase gene (POL), the first exon of the terminase gene (ORF62), the second exon of the terminase gene (TERM), and the junction between the polymerase and exon 1 (POLIN-TERM). Out of the four primer pairs, we only managed to amplify a product using the TERM and POLIN-TERM (Figure 5A,B), despite numerous attempts. Unfortunately, the amplification of the junction of the DNA polymerase and exon 1 was not very specific and no fragment was amplified to the correct size, 6776 bp, so they were, therefore, not sequenced. The sequencing of the TERM fragment verified the presence of EsHV1, being 98% identical to the published sequence of EsHV1 terminase exon 2.

### 3.2. Next-Generation Sequencing

MiSeq Illumina sequencing in combination with MinION nanopore sequencing was carried out directly on the clinical specimens. The read coverage, as reported by SPAdes, was roughly 70. For the purpose of the present study, a ca. 27,000 nt long fragment was extracted, encompassing the DNA packing terminase and DNA polymerase genes (accession PQ650602; sequence reads are available at the sequence reads archive (SRA) at GenBank with accessions SRR316631688 and SRR316631688). The sequence was very divergent compared to other related alloherpesviruses. For both the polymerase (WWU01563) and the terminase (WZD86159), the most similar amino acid sequence originated from white sturgeon herpesvirus 2 (official species designation: *Ictavirus acipenseridallo 2*), with amino acid identities of 29.5% and 39.5%, respectively. On the other hand, the previously partly sequenced terminase and polymerase genes of a North American Esocid herpesvirus [15] were closely similar and displayed amino acid identities of 98.2% (1062 aa of polymerase, KX198667) and 97.4% (1993 aa of terminase, KX385272). The other proteins of North American Esocid herpesvirus, for which the sequences were determined, also displayed high amino acid similarity to the Swedish virus. Using the numbering of accession KX198667, the amino acid identity values were 99.4, 98.8, and 100% for ORF59 (major envelope protein), ORF60 (Allo60), and ORF61, respectively.

### 3.3. Phylogeny of EsHV1

**Figure 6:**
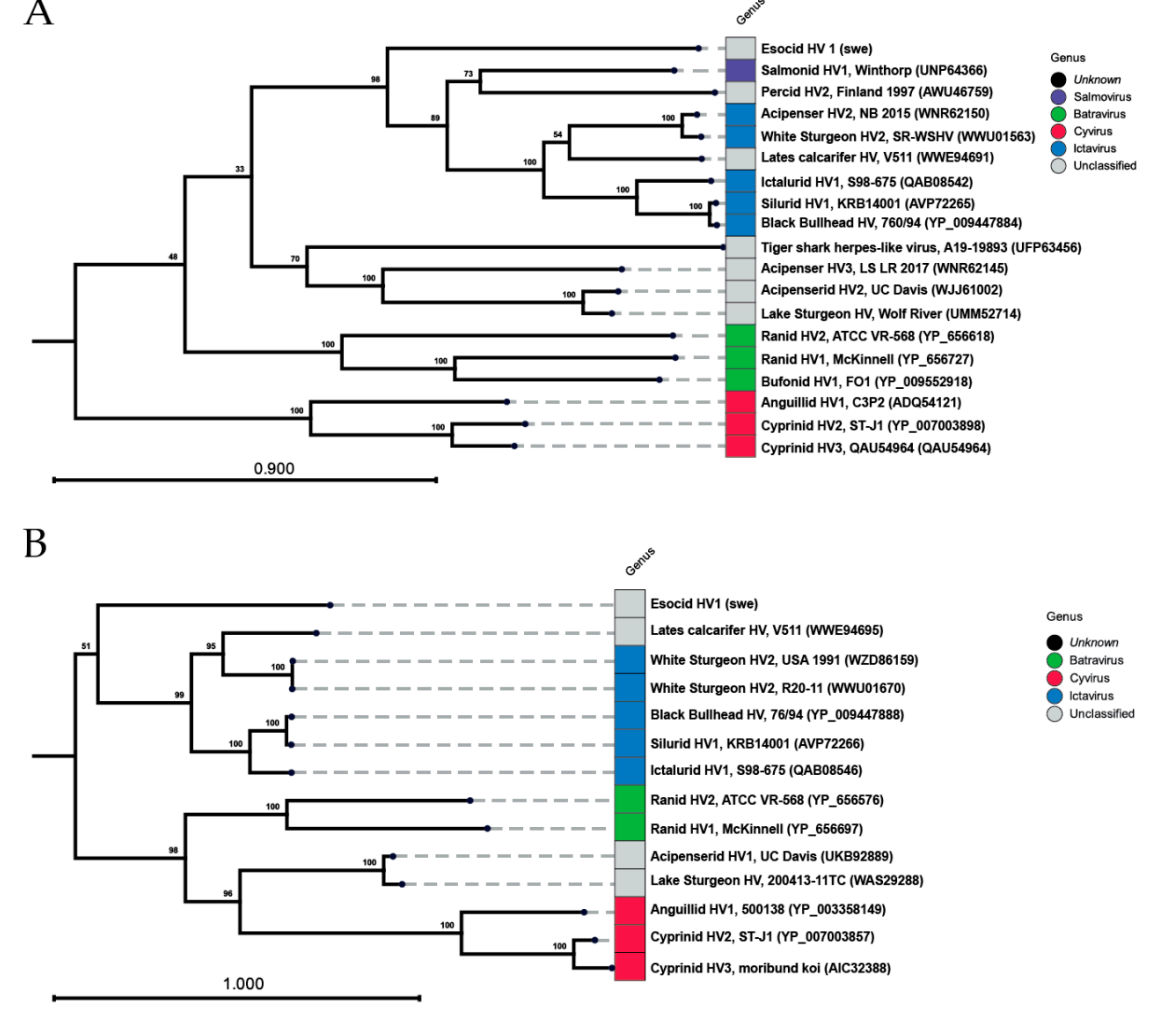
Maximum likelihood phylogeny of the EsHV1-swe: (**A**) ca. 950 amino acids of the DNA polymerase and (**B**) DNA packing terminase comparing related and previously characterized alloherpesviruses infecting fish and amphibians. The Swedish isolate is highlighted in red. The genera as currently defined are shown with different colors. Virus names and isolate names are given when available; otherwise, country and year of collection are shown. Accession numbers for included sequences are given within parentheses. The bootstrap values of 100 replicates are shown at the nodes.

The family of *Alloherpesviridae* varies significantly in genome size and exhibits high genetic diversity. In addition, most of the gene products are uncharacterized and are only automatically annotated and not functionally investigated. Therefore, when investigating phylogentic relationships, usually, the DNA polymerase, terminase, and helicase are used, as they are usually the most well-conserved genes between viral species. Figure 6 shows the maximum likelihood phylogeny of ca. 950 amino acids of the DNA polymerase (A) and the full sequence of the DNA packaging terminase (B). A fragment of the DNA polymerase sequence was used to allow for comparison with the only significantly characterized representative of the *Salmovirus* genus, *Salmonid herpesvirus 1*, which has a shorter polymerase sequence, possibly because a second exon does not exist or has not been identified. For the polymerase, the *Cyvirus* genus forms a single clade, while the remaining genera are grouped into three clades formed by the batraviruses; the hitherto-unclassified *Acipenserid herpesviruses 1* and *3* together with the likewise unclassified tiger shark herpes-like virus; and a large clade formed by the *Ictavirus* genus and the single salmovirus together with a few unclassified alloherpesviruses, including the Esocid herpesvirus presented in the present study. However, the Esocid herpesvirus is distinctly different, and no other virus in the present data set can be singled out as the closest relative with certainty. The terminase tree displays roughly the same picture, but with the difference that the batravirus and the acepenserid viruses are now associated with each other in a clade separate from the ictaviruses and the unclassified Esocid herpesvirus (this study) and Lates calcarifer herpesvirus. It should be noted that the bootstrap support for the most ancient branches is weaker due to the high sequence diversity of these viruses. Thus, for the terminase, the Esocid herpesvirus is most closely associated with the ictaviruses, albeit on a single branch and with low sequence homology with the ictaviruses.

**Figure 7:**
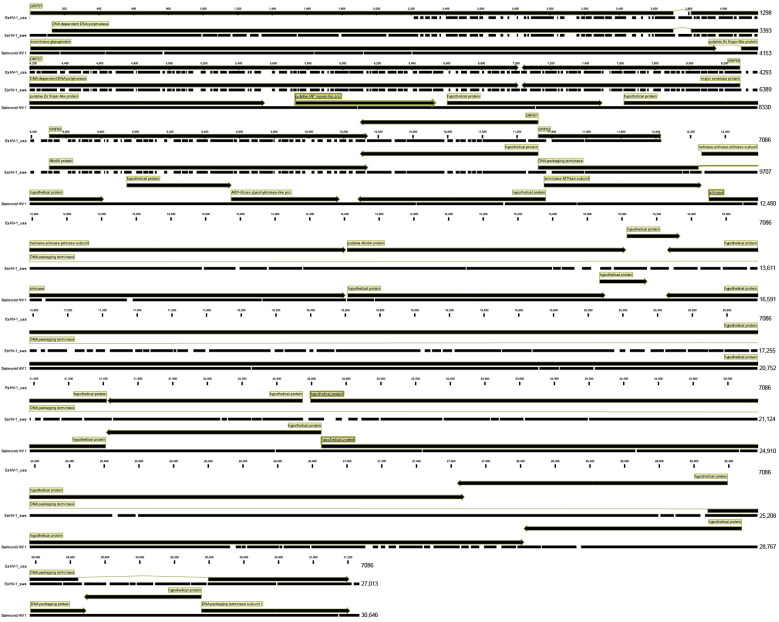
The North American (EsHV-1_usa) and Swedish (EsHV-1_swe) *Esocid herpesvirus 1* with accessions KX198667 and PQ650602 aligned together with the corresponding region of Salmonid Herpes 1 (accession OK337613). Coding region annotations are as given in GenBank.

In Figure 7, the predicted coding regions of the two Esocid viruses and those of Salmonid herpesvirus in the corresponding region are shown aligned. It is clear that the predicted protein-coding regions are identical for the two Esocid viruses, with the single exception being that the starting codon for the polymerase appears to be more upstream for EsHV-1_usa. However, this is at the end of the EsHV-1_usa sequence, and it is unknown which coding regions would be predicted with more sequence data available. The *Salmonid herpesvirus 1* DNA polymerase is located elsewhere in the genome, but from the hypothetical protein corresponding to ORF61 of EsHV-1_usa, there is a close correspondence between the predicted coding region for EsHV-1_swe and *Salmonid herpesvirus 1*. For example, the helicase–primase primase subunit is roughly of the same length and is located at the analogous position, as well as the three terminase exons, although not annotated as spliced together for the Salmonid herpesvirus.

## 4. Discussion

Herpesviruses are common pathogens in all living organisms. The order *Herpesvirales* contains three families of herpesviruses named *Malocoherpesviridae*, *Orthoherpesviridae,* and *Alloherpesviridae*. To date, there are 133 different species of herpesviruses listed by ICTV [26,27]; 118 of these are members of *Orthoherpesviridae* affecting avian, reptilian, and mammalian hosts. Given the number of herpesviruses affecting terrestrial living organisms, it is not surprising that molluscs (*Malocoherpesviridae)*, crustaceans (*Alloherpesviridae*), and fish (*Alloherpesviridae*) are also affected by herpesviruses. In fish alone, there are currently 68 complete sequences of herpesviruses which have been isolated [27]; however, it is important to point out that not all of them have been reviewed and approved by ICTV. Thus, the true numbers of herpesviruses in fish as well as the diversity of *Alloherpesviridae* are likely at least as complex as its terrestrial counterpart.

During the annual surveillance of the spawning in Lerviksbäcken, Kalmarsund, Sweden, a male pike with the blue spots characteristic of EsHV1 infection was caught. The pike was euthanized and samples were collected for DNA extraction, electron microscopy, and histology. Unfortunately, no samples were collected for culturing, as it was impossible to transport the sample to the laboratory for culturing in a timely matter. In addition, skin was not included in the tissue fixed in formalin for histology, and instead, the histological investigation had to be performed on ethanol-fixed tissue intended to be used for DNA extraction. The pike was in overall good condition, and the infection did not seem to affect the feeding behavior. Histological investigation supported this as there was extreme vacuolization of the liver, showing massive energy storage in the hepatocytes. Further, none of the internal organs showed signs of a systemic viral infection, which suggests that the EsHV1 infection was limited to the skin. Infected areas of the skin showed a thickening of the epidermis and the presence of large basophilic structures. These large structures were likely hypertrophied infected epidermal cells based on the similarity to other epithelial cells infected with herpesvirus (e.g., cod herpesvirus (GaHV-1) in gills [25], Figure 3) and the known EsHV1 etiology of “blue spot disease” [15,16]. Some of these structures contained smaller cells which could have been lymphocytes fighting off the infection. In our histological investigations, we could not see any evidence of progression or regression of the infection. It would, however, be interesting to further investigate the material to look for signs of a latent infection, as that could suggest that an EsHV1 infection could become reactivated under certain conditions. The TEM investigations very much agreed with previously published papers [15,16] with the presence of three different types of particles (A-, B-, and C-particles) budding from the nuclear membrane. In the infected sections of the skin, numerous viral particles could easily be identified, suggesting a major infection by the virus with a significant production of progeny viral particles. Viral particles were often, but not always, associated with large vacuoles or a network of connected vacuoles, which has previously been reported for alloherpesviral infections in fish [28].

In common with earlier investigation on, for example, the *White Sturegeon herpes virus 2* (accessions: WWU01670 and WWU01666), we find the sequence data to be compatible with the polymerase and terminase composed of two and three exons, respectively (see accession PQ650602). Both proteins are very divergent compared to other alloherpesviruses and form a distinct branch (Figure 6). However, both proteins are most closely associated with the Ictaviruses and, in both cases, display the highest amino acid identity with *White Sturgeon herpesvirus 2*. This result is in accordance with the previously partially sequenced polymerase of an *Esocid herpesvirus 1* sampled in Lower Mud Lake, Dane County, WI, USA [15]. The amino acid identity between the North American and Swedish viruses is high for both the polymerase (98.2%) and terminase (97.4%). However, there are significant differences at the DNA level, which is highlighted by the fact that only one out of the four published primer pairs for EsHV1 detection worked on our isolate. After aligning the primer sequences used by Freitas et al. [15] to our EsHV1 sequence, it was evident why two out of the four assays shared no, or very limited, homology to our sequence. With the observed differences and the placement of EsHV1 in different clades of *Alloherpeviridae,* it would be interesting to obtain additional sequence data and to compare the full genome of EsHV1 to other fully sequenced alloherpesviruses.

To our knowledge, there are no public records held in Sweden, or anywhere else in Europe, over identified abnormalities in pike, likely because the species is typically not included in biomonitoring programs due to being captured representatively in standardized surveys methods such as gill-nets [4]. In this study, we are, for the first time in mainland Europe, identifying EsHV1 in pike. The effects of EsHV1 infections, and possible recurring infections, in a rapidly declining pike population such as the one in the Baltic Sea are difficult to estimate. It is plausible that viral infections can contribute to the decline by affecting the fitness of pike, but, unfortunately, very little is known about how viral infections affect the survival and health of pike. During our survey, 81 pikes were caught over three days, and in addition to the EsHV1-infected pike, nine pike with lymphosarcoma, a disease caused by a type C-retrovirus, were identified [9,11,29]. It is, however, important to point out that we did not perform any assays to verify that these tumors were caused by retroviruses, but assumed so given the evidence in the literature. In total, 10 out of 81 pike were visibly affected by a virus infection. Not surprisingly, this number is likely low, as both herpes- and retroviruses are known to cause latent infections [30,31]. Our observation from Lervik is somewhat reflective of an old study from the end of the 1970s in Heming Lake, Manitoba, Canada, by Yamamoto et al. [10], but significantly lower compared to the 11% average in selected Wisconsin lakes [32]. Yamamoto found the prevalence of sarcoma and presumably EsHV1 to be 1.19% and 1.31%, respectively, a similar prevalence for EsHV1 to ours at 1.2% (1 out of 81), even though our sample size was considerably smaller. Of note is that we noticed a much higher prevalence of lymphosarcoma at 11.1% (9 out of 81 pike). Whether the presence of viral infections in pike is a contributing factor to the diminishing population in the Baltic Sea must be further investigated to ensure the survival of the species given its pivotal role in the ecosystem.

## Data Availability

The original data presented in the study are openly available in GenBank (https://www.ncbi.nlm.nih.gov/genbank/, accessed on 1 November 2024) accession number PQ650602).
